# Seasonal Sensory Evaluation of Low Commercial Value or Unexploited Fish Species from the Portuguese Coast

**DOI:** 10.3390/foods9121880

**Published:** 2020-12-17

**Authors:** Frederica Silva, Ana M. Duarte, Susana Mendes, Elisabete Magalhães, Filipa R. Pinto, Sónia Barroso, Ana Neves, Vera Sequeira, Ana Rita Vieira, Leonel Gordo, Maria Manuel Gil

**Affiliations:** 1 MARE—Marine and Environmental Sciences Centre, Polytechnic of Leiria, Cetemares, 2520-620 Peniche, Portugal; frederica.g.silva@ipleiria.pt (F.S.); ana.c.duarte@ipleiria.pt (A.M.D.); filipa.gomes@ipleiria.pt (F.R.P.); sonia.barroso@ipleiria.pt (S.B.); 2 MARE—Marine and Environmental Sciences Centre, Faculdade de Ciências, Universidade de Lisboa, 1749-016 Lisbon, Portugal; eli_magalhaes_silva@hotmail.com (E.M.); amneves@fc.ul.pt (A.N.); vlsequeira@fc.ul.pt (V.S.); arivieira@fc.ul.pt (A.R.V.); lsgordo@fc.ul.pt (L.G.); 3 MARE—Marine and Environmental Sciences Centre, ESTM, Polytechnic of Leiria, Cetemares, 2520-620 Peniche, Portugal; susana.mendes@ipleiria.pt; 4 Departamento de Biologia Animal, Faculdade de Ciências, Universidade de Lisboa, 1749-016 Lisbon, Portugal

**Keywords:** sustainability, sensory characterisation, discarded fish, seafood, fishing, season

## Abstract

Overfishing is increasing over time, and according to FAO (Food and Agriculture Organization), about one third of the world’s fish stocks are now overfished. Thus, diversifying the target species is essential for fisheries sustainability contributing to improve resource-efficient processes. Non-target species can be valuable resources for the development of new food products. However, those species are scarcely studied, and it is of high importance to trace their seasonal sensory profile as a first step towards their valorisation. Therefore, in this study, seasonal influence on sensory properties of five low commercial value or unexploited fish species, namely *Trachurus picturatus* (blue jack mackerel), *Spondyliosoma cantharus* (black seabream), *Trigla lyra* (piper gurnard), *Serranus cabrilla* (comber) and *Capros aper* (boarfish), was assessed in order to identify the most favourable season for catching each species. Fish samples were assessed by a panel of 16 semi-trained assessors for sensory attributes previously identified. The evaluation takes place every 2 months. Statistical differences were reported between attributes and seasons for all species, except for *T. lyra*, which did not present any difference in its sensory attributes throughout the year.

## 1. Introduction

By-catches (the catch of species for which there is no direct effort) and discards (the part of the by-catch that is not used and is, therefore, thrown overboard) are a global phenomenon resulting from fisheries and have been of great concern for all stakeholders in the sector, such as industries, fishermen or scientists [1]. Since the early 1980s, some studies have shown that discards have reached 38 million tonnes (representing 40% of the total catch) but, as a result of further research and restrictions, they fell to around 27 million tonnes by 2014 [2].

By-catches occur because some fishing gears are less selective than others (for example, longline is more selective than gillnets and trawl), catching many more species than those targeted by the fishery [3]. This may lead to discards of many fish species which may be due to: (a) technical reasons for marketable species (e.g., onboard storage capacity, bad weather), (b) economic reasons (e.g., species with no or low commercial value, inexistence of a ready market for certain species, damage or poor quality of fish), (c) legal and administrative reasons (e.g., minimum legal sizes of marketable species, commercial fishing quotas already exceeded, unauthorised fishing licenses) and (d) biological/ecological reasons (e.g., patterns of distribution of species which in turn conditions the directed fishery for one or multiple species) [1,4,5]. In this context, challenging aspects are therefore: (i) understanding which species of low or no commercial value have an added potential for their nutritional value, to be used as food, and (ii) the progressive reduction of fish discards, developing alternatives for valorisation of those species, aiming of maximising the return on fishing captures and contributing to long-term environmental, economic and social sustainability. 

The Atlantic Ocean is a valuable source of fish, which is a high-protein, low-fat food that provides a range of health benefits. As highly reported, depending on species, seafood can be an important source of proteins of high biological value, rich in polyunsaturated fatty acids of the omega-3 series, a source of vitamins (such as vitamin D) and minerals (such as Se, P and Ca), and essential amino acids [6]. Besides this, it is well known that fish plays an important role in global food security and nutrition, contributing essential nutrients, which are important to combat malnutrition throughout the world, especially for coastal populations in many undeveloped and developing countries. However, we live in a world of limited biological resources and, consequently, improving the efficiency of fish value chains to reduce losses and waste, in an effort to improve access and affordability to all, is of utmost importance and is essential for fisheries’ sustainability contributing to improve resource-efficient processes and circular economy [7].

What is meant by “commercial species” and “low commercial values species”? In the beginning of this century, the authors of Reference [8] collated information from European laboratories and government agencies on commercial species in Europe, considering commercial species as “one that is subject to a contemporary local or regional fishery in a certain period of their life (whether as a target species, or a by-catch which is landed)”. In total, 162 fish species from the Atlantic area were catalogued, and generally speaking, there is a tendency for an increase in the number of commercial species from northern to southern areas, with a mean number of 18.7 species from Baltic, North Sea and Celtic Sea areas to 44.7 species in the Iberian Peninsula [1]. This increase in the number of commercial species is due not only to the higher biodiversity in southern areas but also to the more diverse fish-eating habits in both Portugal and Spain [9]. This larger number of commercial species with different commercial value, associated with the traditional feeding habits of Portuguese consumers, established different categories of market prices for fish species, with the less known and the smaller species traditionally having a lower commercial value. Despite its costal location, the two most consumed species in Portugal are imported (the cod and the salmon) and sold at ca 10 € per kilo, a price above other very popular and local species, like hake, octopus and scabbardfish, sold between 3.5 and 6.5 € per kilo in the first auction [10]. 

Among species with low commercial value, the selected species *Trachurus picturatus* (blue jack mackerel), *Spondyliosoma cantharus* (black seabream) and *Trigla lyra* (piper gurnard) are particularly important. Firstly, for their landings (e.g., *T. picturatus* can reach 2800 t yr^−1^ in the last decade), but also due to the price they can achieve in the first auction that, during this study, ranged between 1.37 and 2.06 € per kilo.

Among species without commercial value, *Serranus cabrilla* (comber) and *Capros aper* (boarfish) are particularly abundant, the latter being species object of concern by the International Council for the Exploration of the Sea (ICES) in terms of its sustainability, given the high catches taken, for example, in the Celtic Sea [11]. In fact, species that have lower or even no interest to be used in human consumption are used for non-food purposes like pet food, or as raw material for direct feeding in aquaculture, livestock and fur animals. In 2018, 22 million tonnes (12% of the global fish production) were in this category [8], and for these reasons, ICES has recommended monitoring programmes for stock management, as well as the acquisition of biological information on these species [11]. *S. cabrilla* and *C. aper* are included in the species that are caught as by-catch and have no commercial value in national waters, being discarded on board, unlike other European countries. In Portuguese waters, *S. cabrilla* is one of the most important species in terms of rejection of several fisheries, namely gillnets and trawls, while *C. aper* is among the ten most important species in terms of abundance, being caught as bycatch in both crustacean and fish trawl fishing [1,12,13,14].

The referred species are some examples of low or no commercial value, but due to their mentioned characteristics, they can provide an alternative to the species normally caught/consumed (species of high commercial value) and contribute to decreasing overfishing.

One of the ways to contribute to marine ecosystem maintenance is to shift the consumer demand towards more sustainable seafood products, to reduce the overexploitation of most consumed fish species [15]. However, to achieve that behaviour shift, it is crucial to understand if discarded or low commercial value fish species present properties that stimulate their purchase in detriment of others. Nevertheless, the valorisation of underutilised species can be achieved not only by direct human consumption or added value bioproducts for the food sector, but also by bio compounds’ extraction (e.g., enzymes, collagen and gelatines; pigments) for different applications, including in medical and pharmacological sectors, meals and silage from marine species and leather [3,16]. Thus, to promote these species, it is necessary, among other factors, to understand their seasonal sensory properties, as well as the most favourable season for their catch. Therefore, the consumption of these discarded fish can be enhanced, which promotes their commercial valorisation. In this sense, and among the different sensory analysis methods, the use of a semi-trained panel of tasters can be used as an instrument to assess the magnitude of sensory attributes. Thus, this investigation aims to characterise five fish species, namely blue jack mackerel, black seabream, piper gurnard, comber and boarfish, based on their sensory properties, as well as to outline the relationship between fish attributes and season of the year, enabling the selection of the best season for their capture.

## 2. Materials and Methods

### 2.1. Fish Samples Preparation 

The species under study (blue jack mackerel, black seabream, piper gurnard, comber and the boarfish) were captured in the Portuguese coast during 2019 and collected every 2 months (in January–February, March–April, May–June, July–August, September–October, November–December), from the Peniche fishing harbour, and kept at 4 °C on the capture day. The samples were packed and stored at −20 °C in polyethylene bags until further analysis (7–9 days). On the experiment day, the fish previously thawed overnight (4 °C) were cut into fillets with skin and steam-cooked (without addition of salt or oil) at 100 °C for 10 min using a kitchen robot (Bimby, Vorwerk, Thermomix 31-1, Wuppertal, Germany). 

### 2.2. Sensory Analysis

Sixteen selected and semi-trained assessors undertook the sensory descriptive analysis on five fish samples. Performance is a measure of a panel or evaluator’s ability to make valid attribute assessments of the evaluated products. It can be monitored at a certain point in time or tracked over time. Performance comprises the ability of a panel to detect, identify and measure an attribute, use attributes in a similar manner to other panels or assessors, discriminate stimuli, use a scale appropriately, repeat its own results and reproduce the results of other panels or assessors [17]. The generation of the descriptors was based on the identification of their main sensory descriptors that were selected in our previous study [18], using CATA (Check-all-that-apply) methodology and a semi-trained panel, formed as previously reported [18]. In the CATA method, the panel members select the descriptors that best describe the test product from a given list. Those main sensory descriptors generated a list of attributes that was used by the panellists in the present study, to evaluate each fish species every 2 months throughout the year. The checklist was divided into four categories: odour, appearance, taste/flavour and texture (Table 1). For each sample, the panel members were instructed to mark on the checklist the intensity degree (from 1 to 5, where 3 is the ideal, 1 is absent and 5 too many) perceived for each descriptor. 

All tests were conducted in accordance with ISO (International Organization for Standardization) standards ISO 8586 [19] and ISO 11132 [20] in accordance with the International Organisation for Standardisation. Each sample was coded by three random digits, and cutlery, napkins and glass cups of mineral water were provided, as well as rusks to clean the palate between the samples. The panellists evaluated the cooked fish fillets in individual sensory booths in a sensory analysis laboratory (with temperature and lighting control). 

### 2.3. Statistical Analysis

A one-way analysis of variance (ANOVA) was performed to assess the statistically significant differences between months for each species descriptor. Data normality and homogeneity of variance were also validated, and multi-comparison tests were performed by the Tukey or LSD (Least Significant Difference) tests [21]. The use of the ANOVA proved to be adequate, as it is sufficiently robust, in order to withstand violations of the interval data assumption and moderate skewing [22,23]. When the ANOVA assumptions were not met, the non-parametric Kruskal–Wallis test was performed followed by the Games–Howell multi-comparison test. The use of the Kruskal–Wallis test showed to be appropriate since it allows comparing distributions of two or more at least ordinal variables observed in two or more independent samples [24]. In order to compare the sensory pattern that is common for each species throughout the year, a matrix (input data) was constructed with the mean classifications of each month (rows) by descriptors (columns), followed by a principal component analysis (PCA) [25] to reduce the dimensionality of the data, but maintaining the relevant information contained therein [26]. The PCA procedure was performed on the covariance matrix, since the sensory scales are the same for all attributes [27]. The principal components (PC) are calculated by linear combination of original variables and adequately represent the original data [28]. For ANOVA, IBM SPSS Statistics 26 (Copyright IBM Corp. © 1989–2019, Armonk, New York 10504-1722, USA) was used. For the PCA, Canoco for Windows 4.5 software (Copyright Petr Smilauer © 2012–2019, Ithaca, New York 14850, USA) was used [25]. All results were considered statistically significant at the 5% level (i.e., whenever *p*-value < 0.05).

## 3. Results and Discussion

The complex nature of sustainable seafood consumption is dependent on motivational variables, such as intentions that are preceded by an attitude which are mainly formed through beliefs about taste, distaste, nutritional value, ease of preparation, familiarity and freshness [29]. Therefore, it is important to describe the sensory pattern of unexploited fish, in order to identify their market potential as a substitute of commercial species. 

In the present study, the sensory data over the year for each fish species allowed the identification of their main sensory characteristics for direct consumption and/or application in a fish products formulation. These data will be discussed according to season for each species.

### 3.1. Blue Jack Mackerel

**Figure 1:**
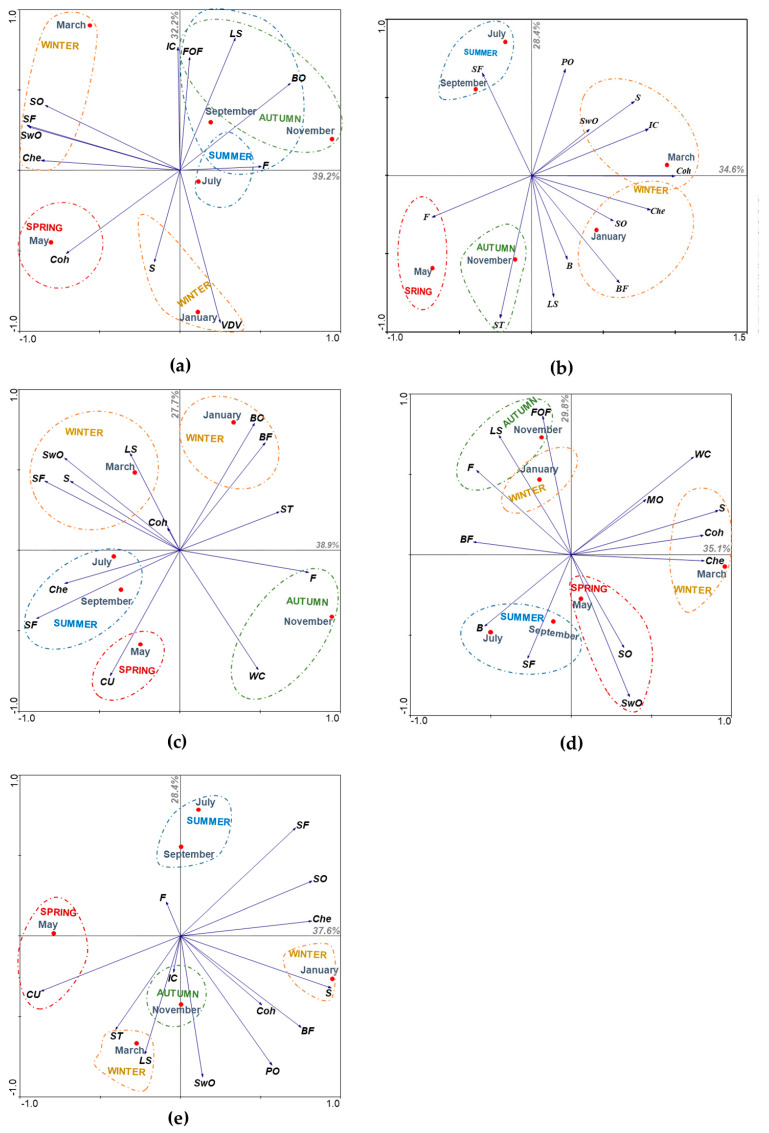
Principal Component Analysis (PCA) for sensory descriptors among species throughout the year for (**a**) blue jack mackerel, (**b**) black seabream, (**c**) piper gurnard, (**d**) boarfish and (**e**) comber. Legend: SO: sea odour; SwO: seaweed odour; BO: butter odour; VDV: visible dark veins; IC: ivory colour; LS: laminar structures; FOF: fish oil flavour; F: fat; SF: sea flavour; S: stiffness; Che: chewability; Coh: cohesion; PO: potato odour; B: brightness; BF: butter flavour; ST: sweet taste; CU: colour uniformity; WC: white colour; MO: metallic odour.

Blue jack mackerel sea odour, butter odour and ivory colour were statistically different bimonthly (ANOVA, *p*-value = 0.010, 0.037 and 0.007, respectively), as well as seaweed odour and stiffness (Kruskal–Wallis, *p*-value = 0.000 and 0.003, respectively). It is notable that the main differences occurred between winter (January and March), spring (May) and autumn (November) in relation to other seasons, although both winter months (January and March) also presented some differences between them (Table 2 and Table 3). The highest ratings were reported in late winter (March) for sea and seaweed odour as well as for colour ivory, while they were achieved in the autumn (November) for butter odour and in the spring (May) for firmness. It should be noted that such high ratings, in all species in the present study, did not exceed the limit considered “ideal” (classification 3) by the panellists. Regarding PCA results, the two main components (PC1 and PC2) together explained 71.4% of the variability in blue jack mackerel descriptors (Figure 1a). The first component PC1 explained 39.2% of the sensory variability and is characterised by sea odour and flavour, as well as by seaweed odour and chewability (Figure 1a). These descriptors correlate in a positive and intense way, describe the sensory pattern at the end of winter (March) and are opposed to fat (Figure 1a). Although these are the descriptors that most describe the blue jack mackerel captured at the end of winter (March), cohesion and stiffness also showed some expressiveness, but with less preponderance than in spring (May) (Figure 1a). Thus, it can be concluded that the descriptors associated with the end of winter (March) were evaluated more positively than in autumn (November) (Figure 1a). On the other hand, in the beginning of winter (January), blue jack mackerel is associated with visible dark veins and stiffness, the latter with less expression (Figure 1a). Such descriptors were evaluated more positively at the end of winter (March), compared to the ivory colour, fish oil flavour and laminar structures, where this opposition characterises the second component PC2 which explained 32.2% of the sensory variability (Figure 1a). The summer has a low differentiating character in the sensory pattern of blue jack mackerel, although at the end of the season (September), this species was especially associated with fish oil flavour, laminar structures and butter odour (Figure 1a). Finally, it appears that blue jack mackerel cohesion had higher scores in the spring compared with its laminar structures and butter odour, proving to be opposite to the end of summer (September) and autumn (November) (Figure 1a). Considering these results, it appears that when blue jack mackerel is caught in the late winter (March), in autumn (November) or in late summer (September), it reveals a greater number of sensory descriptors compared to the other seasons (Figure 1a). It is important to note that when the objective of applying this species is to obtain the maximum fat content perceived by the consumer, the blue jack mackerel capture should be carried out, especially in the beginning of the summer (July) (Figure 1a). Therefore, this species revealed high potential to be marketed as fresh, especially at the end of winter (March), where the sea and seaweed flavour are more prominent. These attributes are considered as indicative of the fish freshness and, therefore, more appealing to the consumer. On the other hand, according to these findings, blue jack mackerel can also be applied in processed fish products, such as fish burgers, where the firmness and cohesion of the meat are important factors in the product integrity maintenance, with the most favourable season for the catch being spring (May). 

### 3.2. Black Seabream 

Black seabream data reveal statistical differences in seaweed odour (ANOVA, *p*-value = 0.018) and sweet taste (ANOVA, *p*-value = 0.002), with the highest ratings being recorded in late winter and autumn, respectively (Table 2 and Table 3). These differences were reported in seaweed odour between the winter months (January and March) as well as between the end of this season (March) compared to autumn (November) (Table 2 and Table 3). Likewise, the black seabream sweet taste showed statistically significant differences between autumn (November) and summer (July and September) (Table 2 and Table 3). According to PCA (Figure 1b), the first factorial plan explained 63.0% of the total data variability, where 34.6% is explained by PC1 and 28.4% by PC2. PC1 is mostly characterised by cohesion, followed by the intense and positive relationship between ivory colour, stiffness and seaweed odour (Figure 1b). Although cohesion is the most preponderant descriptor in late winter (March), this season is also associated with ivory colour, stiffness and seaweed odour, which are opposite to black seabream fat content that revealed higher rates in the spring (January and March) (Figure 1b). The beginning of winter (January) has a low differentiating character in black seabream sensory pattern, revealing some association with butter flavour, sea odour and chewability (Figure 1b). These descriptors reveal an opposite behaviour with sea flavour, which was evaluated more positively in the summer (July and September) (Figure 1b). Autumn (November) also has a low differentiating character in black seabream sensory pattern, revealing some association, especially with sweet taste, which opposes with potato odour, corresponding this opposition to the characterisation of the second component PC2 (Figure 1b). Therefore, when black seabream is captured in winter (January and March), it has descriptors classified more positively and in greater numbers compared with the other seasons, making this season the most favourable for catch (Figure 1b). On the other hand, when a higher level of fat perceived by the consumer is desired, the catch should be carried out in the spring (May) (Figure 1b). Due to the physical similarity of black seabream with the common species *Sparus aurata* (gilt-head seabream), its commercialisation will be facilitated, avoiding the need for transformation for its valorisation. Thus, considering the ivory colour as well as the attributes of freshness such as the seaweed odour, the end of winter (March) is considered the most favourable season for capture. On the other hand, due to the ivory colour of its meat, it can also be sold in substitution of *Merluccius merluccius* (hake) used in fish sticks, as well as in frozen fillets or loins form. Thus, considering stiffness and ivory colour as major factors for application on frozen fish sticks, fillets or loins, the most favourable season for the capture of this species will also be the end of winter (March).

### 3.3. Piper Gurnard

For piper gurnard, no meaningful descriptors were reported (*p*-value > 0.05). Thus, for this species, the descriptors evaluated did not suffer a significant effect of seasonality. PCA analysis throughout the year is presented in Figure 1c, where the first factorial plan explained 66.6% of the descriptors’ total variability, divided in 38.9% for PC1 and 27.7% for PC2. PC1 is characterised by the opposition between laminar structures and white colour, where the first descriptor presents greater preponderance in piper gurnard sensorial pattern at the end of winter (March) (Figure 1c). At this time of the year, piper gurnard presents an intense and positive relationship between seaweed odour, stiffness and sea flavour, although they have little preponderance in the sensory pattern in the late winter (March) (Figure 1c). However, at the beginning of winter (January), piper gurnard is associated with butter odour and flavour, which are opposed to its colour uniformity that is associated when this species is captured in the spring (May) (Figure 1c). PC2 is characterised by the opposition between the summer (July and September) and autumn (November) (Figure 1c). In the summer, the piper gurnard is characterised by chewability and sea flavour, related in an intense and positive way (Figure 1c). In autumn (November), the sensory pattern of piper gurnard is characterised by fat and white colour, although the latter with less expression (Figure 1c). When this species is captured in late winter (March), in summer (July and September) and in spring (May), a greater number of descriptors are perceived compared to autumn (November) and the beginning of winter (January) (Figure 1c). The commercialisation of this species as whole in fresh fish, may be hampered by the presence of spines in the operculum area and ventral fin, making the preparation process (such as evisceration) more laborious, discouraging the consumer to buy such a product. Therefore, because piper gurnard was not considered a species with firm meat and the fillets are thinner and less appealing, it is considered that the sale as a fillet may discourage its commercialisation. Considering the butter odour and flavour generally appreciated in snacks, the piper gurnard fillets can be dehydrated after frying with a consequent increase of the fillet’s firmness, allowing the crispy texture so valued in this type of product. Such attributes are especially found in the beginning of winter (January) and it is recommended to capture the piper gurnard in this season. Additionally, with the development of this type of snack, the consumption of fish will be promoted, resulting in a healthier snack that can also be added to salads, nutritionally enriching this type of product.

### 3.4. Boarfish

Boarfish presented statistical differences in seaweed odour and chewability (ANOVA, *p*-value = 0.041 and 0.048, respectively), as well as in stiffness (Kruskal–Wallis, *p*-value = 0.015). As far as the seaweed odour is concerned, there are differences between the beginning (January) and the end of the winter (March), spring (May) and the beginning of summer (July), where a similar behaviour was reported when these seasons are compared with autumn (November) (Table 2 and Table 3). The highest ratings were obtained in late winter (March) and spring (May) (Table 2 and Table 3). On the other hand, the attributes related to boarfish texture revealed differences mainly between the end of winter (March), where the highest classifications of both attributes were reported, and summer (July and September) and spring (May) (Table 2 and Table 3). According to boarfish PCA results (Figure 1d), the first factorial plan explained 64.9% of the total variability, where the PC1 explained 35.1% and PC2 29.8%. PC1 is characterised by the positive association between cohesion, stiffness and chewability that are opposed to brightness and sea flavour, and especially butter flavour, being the first descriptors associated with the end of winter (March) (Figure 1d). Boarfish caught in the summer (July and September) is characterised by butter and sea flavour, while sea and seaweed odours are associated with spring (May) (Figure 1d). Spring (May) revealed an opposite behaviour with autumn (November), which is characterised mainly by fish oil flavour and, to a lesser extent, by laminar structures and fat, corresponding this opposition to the characterisation of the second component PC2 (Figure 1d). The beginning of winter (January) has a low differentiating character in the sensory pattern (Figure 1d). Thus, it appears that a greater number of descriptors are obtained when the boarfish is captured at the end of winter (March) or autumn (November), compared to summer (July and September) and spring (May) (Figure 1d). Due to the small size of this species, the valorisation through processed products would not be profitable, which is a reason why this should be achieved through its commercialisation as whole and fresh fish. However, due to the unusual boarfish appearance, which may discourage its purchase, its valorisation may go through the heading and sale as fresh breaded and ready to cook. Thus, considering that the consumers are looking for more convenient and practical food products, the boarfish valorisation can be achieved. Considering that the attributes that vary statistically are not the most relevant in this type of product and that in fresh products the most important attributes fall on texture (cohesion, stiffness and chewability) and freshness (seaweed odour and sea odour), the best seasons for capture will be the end of winter (March) and spring (May).

### 3.5. Comber 

Comber sea flavour was the only descriptor with statistical differences through the year (ANOVA, *p*-value = 0.025). Those differences were reported between the winter months (January and March), between the beginning of this season (January) and the beginning of summer (July), with a similar pattern in the spring (May) (Table 2 and Table 3). According to comber PCA results (Figure 1e), the two main components together explained 66% of the descriptors’ total variability. The PC1 explained 37.6%, while PC2 explained 28.4% of total variability (Figure 1e). PC1 is characterised mostly by the opposition between colour uniformity and sea flavour, sea odour and chewability, where the first descriptor has some association with spring (May) (Figure 1e). PC2 is characterised by the opposition between laminar structures, sweet taste and ivory colour with sea flavour, sea odour and chewability, as well as by the opposition between fat and seaweed odour, potato odour, cohesion, butter flavour and stiffness (Figure 1e). Both winter months (January and March) revealed different sensory patterns, where its beginning (January) is characterised by sweet taste and laminar structures, while, at the end, stiffness characterizes comber (Figure 1e). Summer (July and September) and autumn (November) have a low differentiating character in comber sensory pattern, although ivory colour has some association with the latest season (Figure 1e). Therefore, considering that most seasons are not strongly associated with the sensory descriptors, comber capture may be carried out at any time of the year, being aware that the firmness will be “ideal” in the beginning of the winter (January), with subsequent improvement of the sweet taste and laminar structures at the end of this season (March) (Figure 1e). This species has potential for commercialisation as fresh whole fish or for addition to processed products, however it will not be suitable for fillets given the small size of this species. As mentioned, its capture can be carried out at any time of the year, with the exception of products where firmness is the main attribute, and this must take place at the beginning of winter (January). Examples of such products are sausages and hamburgers, where firmness (in association with cohesion) allows the maintenance of product’s integrity before, during and after cooking.

### 3.6. General Discussion

With the analysis of the data as a whole, the species under study revealed high acceptability throughout the year, consequently showing a huge unexploited potential for value adding. In fact, considering as an example a review by Egerton and colleagues about boarfish, there is a large number of potential products and by-products that could be produced with this species, reflecting its great valorisation potential [30]. Those products include fillets, fish mince blocks (for breaded consumables, fish cakes, surimi, etc.), surimi and protein hydrolysates peptides through muscle utilisation [30]. The boarfish skin, not being used, can be utilised for leather, collagen and gelatine and pigments, while viscera can be a source for fish silage, enzymes and oil [30]. Finally, the gonads can be a source of lectins and the headed and gutted fish can be commercialised as pan-fried or breaded [30]. Although nutritional analysis should be carried out to confirm the other species’ potential, they can also be valorised, like boarfish. With the high growth of the world population, it is necessary to develop new policies to ensure the food supply, as well as to support sustainability in socioeconomics and environmental growth in the marine and maritime sector [31]. Thus, the use of discarded fish species must be carried out, for example, through the conversion of these new biomasses in medium–high added value products, such as minced muscle suitable for the preparation of different seafood products with different textures and flavours (e.g., burgers, nuggets or structured fingers) for those specimens above minimum conservation reference sizes (MCRS) [31]. For those specimens captured below the MCRS, their valorisation can be achieved by biotechnological transformation and by-products generated in the recovery of fish mince (heads, skins and bones) for industrial applications as nutraceuticals, pharma, food ingredients and others [31]. However, focusing on direct human consumption, it is important to make known the benefits of consuming discarded species to the consumer that are unknown to him [29]. 

Globally, results allowed perceiving the influence of the season in the sensory characteristics. In addition, the sensory analysis over the year allowed to identify which seasons are more favourable for the capture, considering certain target sensorial characteristics that enable the formulation of a fish product pleasant for the consumer. Despite the fact that most of the fish under study had some heterogeneity in the intensity scored in the sensory descriptors, homogeneity was also found in some cases, revealing that the capture can be carried out at any time of the year without changing the sensory characteristics for each species. 

Despite the importance of the present study, it has some limitations that should be taken into account. Regardless of the seasonal influence on fish sensory descriptors, it should also be considered that there may be some variation in the panel’s judgment due to the different times of the year that this evaluation took place. Therefore, further studies are needed, including consumers’ acceptance studies, to confirm the conclusions of the present work. In addition, the importance of nutritional analysis of the studied species should be emphasised using instrumental methods to corroborate these results and conclusions, in particular those related to fat content.

## 4. Conclusions

The present study allowed to determine the principal sensorial features of five low commercial value or unexploited fish species captured in the Portuguese coast. With these data, it is possible to verify the time of year with the highest number of sensory descriptors perceived by the panellists, which can support the most favourable season for the species capture. In fact, all the species under study revealed statistical differences between attributes and seasons, except for piper gurnard. The lack of heterogeneity of the sensory descriptors throughout the year revealed that piper gurnard can be commercialised at any time of the year, without significant changes in its sensory characteristics. On the other hand, for the remaining species that revealed statistical differences, with the goal of selling them as fresh or processed and the need to be in their best sensory form in order to be appealing to the consumer, the present study described the best time to capture for this purpose. Except for comber, for all the other species, the most favourable season to catch is the end of winter (March) due to the some highlighted sensory attributes. For blue jack mackerel, there is a greater flavour of seaweed and sea at this time of year. For black seabream and boarfish, attributes of better texture and freshness are associated. For comber, there is an ideal firmness at the beginning of winter (January), with an intensification of sweet flavour and laminar structures at the end of this season (March). However, although this is the first study involving seasonal characterisation of these species, more research will be needed to validate these results. It should also be remembered that the species’ valorisation with or without low commercial value does not only involve promoting their fresh sale but can also be achieved in the development of new food products, for cosmetic, pharmacological or medical purposes, and animal feed. 

In the future, the nutritional composition of these species will be assessed, followed by new food products development, that will also be evaluated for their compositional nature. It is also important to analyse the fish consumption habits in Portugal, to verify whether discarded species are already consumed and the consumer’s idea of sustainable habits. In addition, it would be important to perform consumers’ acceptance studies regarding these fish species in order to verify if their preferences meet the panellists’ results.

## Figures and Tables

**Table 1:** Descriptors checklist used for sensory analysis throughout the year for each species [19].

	Species	Blue Jack Mackerel	Black Seabream	Piper Gurnard	Comber	Boarfish
Descriptors						
Appearance		Dark veins	Ivory colour	Colour uniformity	Ivory colour	Brightness
		Ivory colour	Brightness	White colour	Colour uniformity	White colour
		Laminar structures	Laminar structures	Laminar structures	Laminar structures	Laminar structures
Odour		Butter	Seaweed	Sea	Sea	Metallic
		Sea	Sea	Seaweed	Seaweed	Sea
		Seaweed	Potato	Butter	Potato	Seaweed
Flavour		Fat	Butter	Butter	Sea	Butter
		Fish oil	Sweet	Sweet	Butter	Sea
		Sea	Sea	Sea	Sweet	Fish oil
		Fat content	Fat content	Fat content	Fat content	Fat content
Texture		Firmness	Firmness	Firmness	Cohesion	Firmness
		Chewability	Chewability	Chewability	Firmness	Chewability
		Cohesion	Cohesion	Cohesion	Chewability	Cohesion

**Table 2:** Sensory descriptors’ mean range values for each fish species throughout the year.

Fish Species	Descriptor	Mean Values					
		January ^(d)^	March ^(d)^	May ^(a)^	July ^(b)^	September ^(b)^	November ^(c)^
Blue jack mackerel	Sea odour	2.8	3.2	3.1	2.8	2.9	2.8
	Seaweed odour	2.1	3.0	2.9	2.4	2.3	1.8
	Butter odour	2.1	2.5	2.3	2.8	2.7	3.1
	Dark veins	3.3	2.7	3.1	3.1	2.9	3.1
	Ivory Colour	2.2	3.1	2.1	2.9	2.9	2.3
	Laminar Structures	2.6	2.7	2.6	2.6	2.7	2.7
	Fish oil flavour	2.1	2.9	2.8	2.6	2.7	3.1
	Fat Content	2.6	2.5	2.5	2.2	2.3	3.1
	Sea Flavour	2.6	3	2.9	2.7	2.6	2.4
	Firmness	3.2	2.8	3.7	3.5	3.4	3.1
	Chewability	3.2	3.5	3.7	3.4	3.3	3.2
	Cohesion	3.3	3.1	3.3	2.9	3	2.9
Black seabream	Ivory colour	3.1	3.5	2.9	3.1	2.9	2.7
	Brightness	3.4	3.2	3.3	3.2	3.1	3.2
	Laminar structures	3.1	3.3	3.4	2.8	2.9	3.1
	Seaweed odour	2.8	3	2.9	2.7	2.6	2.7
	Sea odour	2.0	3.0	2.4	2.3	2.3	1.9
	Potato odour	2.5	3.0	2.7	2.9	2.9	2.7
	Butter flavour	3	2.9	2.7	2.5	2.7	2.9
	Sweet	2.8	2.7	3.3	2.4	2.4	3.5
	Sea flavour	2.7	2.4	2.6	2.9	2.7	2.3
	Fat content	2.8	2.9	3.1	2.9	2.9	2.9
	Firmness	3.1	3	2.7	3	2.9	2.9
	Chewability	3.3	3.3	2.8	3	2.9	3.3
	Cohesion	3.1	3.4	2.7	2.9	2.8	2.9
Piper gurnard	Colour uniformity	3	3	3.3	3.2	3.1	3.1
	White colour	3.2	3.1	3.4	3.1	3.3	3.4
	Laminar structures	3.4	3.0	3.1	3	2.9	2.7
	Sea odour	2.4	2.7	2.3	2.7	2.5	2.1
	Seaweed odour	2.3	2.3	2.2	2.1	2.2	1.7
Piper gurnard	Butter odour	2.9	2.7	2.3	2.2	2.3	2.5
	Butter flavour	3.4	2.9	2.6	3.1	2.7	3.1
	Sweet flavour	1.9	2.1	2.4	2.4	2.3	1.9
	Sea flavour	3.0	2.7	2.4	3.1	2.7	3.2
	Fat content	2.9	2.9	2.9	2.9	2.7	3.1
	Firmness	3	3.3	3.0	3.1	3.1	2.9
	Chewability	2.9	3.1	3.0	3.3	3.1	2.9
	Cohesion	3.1	2.9	3.1	3	2.9	2.9
Boarfish	Brightness	2.4	2.3	3.0	2.9	2.6	2.7
	White colour	2.7	2.9	2.5	2.4	2.5	2.7
	Laminar structures	2.7	2.3	2.2	2.5	2.4	2.7
	Metallic odour	2.4	3.3	2.5	3.2	3.1	2.7
	Sea odour	1.9	2.9	2.9	2.8	2.6	1.9
	Seaweed odour	2.9	3.9	3.4	3.3	3.3	4.1
	Butter flavour	3.1	2.5	3.1	2.8	2.7	2.7
	Sea flavour	2.6	2.4	2.5	2.9	2.5	2
	Fish oil flavour	2.9	2.7	2.7	2.7	2.5	3.6
	Fat content	3.4	2.6	2.9	3.1	2.5	3.1
	Firmness	2.9	3.3	2.9	2.5	2.6	2.8
	Chewability	3.1	3.3	3.2	2.7	2.8	2.7
	Cohesion	3.0	3.2	3.0	2.7	2.7	2.7
Comber	Ivory colour	2.8	3.1	2.7	2.9	2.6	2.6
	Colour uniformity	2.7	3.1	3.0	2.9	2.9	2.9
	Laminar structures	2.9	3.0	3.1	2.5	2.7	2.7
	Sea odour	2.4	2.1	2.2	2.3	2.2	2.2
	Seaweed odour	2.0	1.9	1.9	1.5	2	2
	Potato odour	2.7	2.6	2.3	2.3	2.6	2.6
	Sea flavour	2.5	1.7	1.8	2.7	2.1	2.1
	Butter flavour	2.9	2.5	2.3	2.2	2.5	2.5
	Sweet flavour	2.5	2.7	2.7	2.	3.1	3.1
	Fat content	2.9	2.3	3.1	2.7	2.7	2.7
	Cohesion	2.9	2.9	2.7	2.7	2.9	2.9
	Firmness	2.9	2.6	2.4	2.6	2.7	2.7
	Chewability	3.0	2.9	2.5	3	2.9	2.9
Legend:		^(a)^ Spring	^(b)^ Summer	^(c)^ Autumn		^(d)^ Winter	

**Table 3:** Sensory descriptors and statistical test value, for those with statistical differences between months/seasons, according to multiple comparison with LSD (Least Significant Difference), Tukey (T) or Games–Howell (GH) statistical tests.

Fish Species	Descriptor	Statistical Test Value	Comparison between Months		*p*-Value
Blue jack mackerel	Sea odour ^LSD^	3.257	March ^(d)^	January ^(d)^	0.007 **
				July ^(b)^	0.007 **
				September ^(b)^	0.023 *
				November ^(c)^	0.007 **
			May ^(a)^	January ^(d)^	0.023 *
				July ^(b)^	0.023 *
				November ^(c)^	0.023 *
	Butter odour ^LSD^	2.490	January ^(d)^	July ^(b)^	0.037 *
				November ^(c)^	0.004 **
			May ^(a)^		0.013 *
	Ivory colour ^LSD^	3.470	January ^(d)^	March ^(d)^	0.009 **
				July ^(b)^	0.026 *
				September ^(b)^	0.043 *
			May ^(a)^	March ^(d)^	0.003 **
				July ^(b)^	0.009 **
				September ^(b)^	0.016 *
			November ^(c)^	March ^(d)^	0.026 *
	Seaweed odour ^GH^	23.622	January ^(d)^		0.002 **
				May ^(a)^	0.012 *
			July ^(b)^	March ^(d)^	0.018 *
			September ^(b)^		0.012 *
			November ^(c)^		0.000 **
				May ^(a)^	0.000 **
	Stiffness ^GH^	18.202	March ^(d)^		0.000 **
				July ^(b)^	0.002 *
				September ^(b)^	0.009 *
			November ^(c)^	May ^(a)^	0.019 *
Black seabream	Seaweed odour ^T^	2.904	March ^(d)^	January ^(d)^	0.025 *
				November ^(c)^	0.014 *
	Sweet taste ^T^	4.230	November ^(c)^	July ^(b)^	0.011 *
				September ^(b)^	0.011 *
Boarfish	Seaweed odour ^LSD^	2.446	January ^(d)^	March ^(d)^	0.029 *
				May ^(a)^	0.019 *
				July ^(b)^	0.042 *
			November ^(c)^	March ^(d)^	0.027 *
				May ^(a)^	0.018 *
				July ^(b)^	0.039 *
	Chewability ^LSD^	2.343	March ^(d)^	July ^(b)^	0.009 **
				September ^(b)^	0.035 *
				November ^(c)^	0.018 *
			July ^(b)^	May ^(a)^	0.035 *
	Stiffness ^GH^	14.138	July ^(b)^	March ^(d)^	0.001 **
				May ^(a)^	0.037 *
			September ^(b)^	March ^(d)^	0.004 *
Comber	Sea flavour ^LSD^	2.725	March ^(d)^	January ^(d)^	0.025 *
				July ^(b)^	0.005 **
			May ^(a)^	January ^(d)^	0.040 *
				July ^(b)^	0.008 **
Legend:	^(a)^ Spring	^(b)^ Summer	^(c)^ Autumn	^(d)^ Winter	

* Results are significant at the 0.05 level; ** Results are significant at the 0.01 level.
